# Supplementary material for: Polarization-multiplexed spatial filtering in a symmetric TiO2 metasurface with an anisotropic van der Waals gap fill
Source: RSC Adv. 2026 Nov 3. Online ahead of print. doi: 10.1039/d6ra07836b (PMC13730192; doi:10.1039/d6ra07836b)
Supplement: Supplementary file 1 [file RA-OLF-D6RA07836B-s001.pdf]

Supplementary Information (SI) for RSC Advances.
This journal is © The Royal Society of Chemistry

Supplementary Information

Polarization-multiplexed spatial filtering in a symmetric TiO_2 metasurface with an anisotropic van der Waals gap fill

Shoumik Debnath and Sudipta Saha

S1 Simulation settings

All results in the manuscript and in this document were computed with the Tidy3D finite-difference time-domain solver.[1] One model and one mesh specification were used throughout, for the normal-incidence spectra and for the oblique-incidence sweeps alike, so the resonance positions quoted in one place and used in another are the same numbers.

The unit cell is periodic in x and y and terminated by perfectly matched layers along z. Oblique incidence uses Bloch boundary conditions taken from the source. The simulation domain is $600 \times 600 \times 3200$ nm^3. The base grid is set by a target of 40 steps per wavelength at a reference wavelength of 890 nm, and a mesh override refines the 60 nm gap to 4.3 nm across its width, giving about fourteen cells across the anisotropic layer. The run time is 2.5 ps with a field-decay shutoff of 10^{-9}. Transmission is recorded on a diffraction monitor placed in the substrate, which returns the complex zeroth-order amplitude on a 0.1 nm wavelength grid between 820 and 960 nm. Amplitude and phase of the transfer function are both taken from that single quantity.

Gap material: α-MoO_3 entered as a diagonal anisotropic medium using the measured constants of Toksumakov *et al.*[2] at 900 nm, with the two short crystal axes in the plane of the flake and the van der Waals stacking axis out of plane: $n_a = 2.382$ along x, $n_b = 2.299$ along y and $n_c = 1.990$ along z. Extinction coefficients $k_a = 4.8 \times 10^{-4}$, $k_b = 3.3 \times 10^{-3}$ and $k_c < 1 \times 10^{-4}$ were included in the separate runs used for Section S4. Bars: TiO_2, $n = 2.35$. Substrate: SiO_2, $n = 1.46$.

For the crosstalk series of Table S3 the crystal tensor was rotated about z by an angle ϕ, producing an off-diagonal component ε_{xy}, and was entered as a full permittivity tensor.

S2 Mesh convergence

Table S1 gives the fitted resonance parameters at three mesh settings. Between 40 and 50 steps per wavelength the TE resonance moves by 0.33 nm and its quality factor by 2.8 per cent; the TM resonance moves by 0.26 nm and its quality factor by 0.2 per cent. Values in the manuscript are quoted at 40 steps per wavelength with a numerical uncertainty of ± 0.3 nm in λ_0 and ± 3 per cent in Q.

No transmittance value above unity occurs at any angle or wavelength in any run reported here.

Table S1: Mesh convergence at normal incidence, corrected tensor.

Steps per wavelength	TE λ_0 (nm)	TE Q	TM λ_0 (nm)	TM Q
30	886.26	51.9	894.36	44.9
40	885.51	50.5	893.84	44.8
50	885.18	49.1	893.58	44.7

As an additional check on the solver, Section 2.3 of the manuscript reports the result of running the same geometry with the calculated constants used before this revision and with the stacking axis placed in the plane of the flake.

S3 Contribution of the gap material to the channel separation

Table S2 reports the resonance positions obtained when the gap of the same structure is filled with different media. The uniaxial entry uses the arithmetic mean of the two in-plane permittivities for both in-plane components and retains the measured out-of-plane value, so the difference between that row and the biaxial row isolates the contribution of the in-plane birefringence.

Table S2: Channel separation for different gap fillings, same geometry throughout. The TE quality factor in the empty-gap row exceeds what a time-domain calculation of this length can resolve and is quoted for completeness only.

Gap filling	TE λ_0 (nm)	TE Q	TM λ_0 (nm)	TM Q	Separation (nm)
Air (empty gap)	862.96	$> 10^4$	877.40	163.3	14.44
Isotropic, $n = 1.46$	876.11	180.9	881.93	142.3	5.82
Isotropic, $n = 2.230$ (rms of n_a, n_b, n_c)	883.95	68.2	900.93	43.7	16.98
Uniaxial, in-plane averaged, c out of plane	885.04	55.1	894.23	43.6	9.19
Biaxial, measured tensor	885.51	50.5	893.84	44.8	8.33

These runs also bear on the naming of the two features. A diagonal tensor aligned with the structural axes preserves both mirror planes of the nanobar pair, so filling the gap does not break a protecting symmetry, and Table S2 shows that an isotropic filler lowers the quality factor in the same way as the measured tensor. Together with quality factors of 50.5 and 44.8, well below the values that motivate the quasi-BIC description, this is the basis on which the manuscript describes both features as Fano resonances.

The isotropic entry at $n = 2.230$ is the root mean square of the three principal indices, which is the index whose permittivity equals the mean of the three principal permittivities. It gives a channel separation of 17.0 nm, larger than the 8.3 nm obtained with the measured tensor. Retaining only the out-of-plane contrast gives 9.2 nm, and adding the in-plane birefringence gives 8.3 nm.

Three contributions can be separated. The empty cell already gives 14.4 nm, so the shape anisotropy of the bar pair and not the gap material sets the scale. Filling the gap moves the separation in either direction according to the index of the filler, to 5.8 nm at $n = 1.46$ and to 17.0 nm at $n = 2.230$. Relative to the isotropic filler of matched index, the anisotropy of the crystal reduces the separation from 17.0 to 8.3 nm, of which the out-of-plane contrast accounts for 7.8 nm and the in-plane birefringence for 0.9 nm, the last being a few times the numerical uncertainty of Table S1.

S4 Absorptive quality factor

Running the corrected tensor with and without the measured extinction coefficients changes the TE quality factor by less than the fit resolution, and the TM quality factor from 44.8 to 44.7. Treating the difference as an absorptive channel,

$$\frac{1}{Q_{\mathrm{tot}}} = \frac{1}{Q_{\mathrm{rad}}} + \frac{1}{Q_{\mathrm{abs}}}, \tag{S1}$$

gives $Q_{\mathrm{abs}} \approx 2 \times 10^4$ for the TM channel. For the TE channel the difference falls below the resolution of the fit, so Q_{abs} is larger still. Both resonances are radiation limited in this band, by more than two orders of magnitude.

S5 Symmetry of the transfer function

The unit cell has a mirror plane normal to x. For illumination tilted in the x–z plane this requires $t(+k_x) = t(-k_x)$, so $H(k_x)$ is an even function of k_x in both amplitude and phase. The computed amplitudes satisfy this

to better than one part in 10^{10}, and simulations run at negative angle reproduce their positive counterparts to the same level.

A first-order spatial differentiator requires $H(k_x) \propto ik_x$, which is odd in k_x. An even transfer function cannot take that form, so a metasurface of this symmetry, illuminated at normal incidence, cannot implement $\partial/\partial x$. This is a property of the symmetry of the cell and does not depend on the choice of optical constants.

The distinction is in the sign structure of the phase and not in its magnitude. A phase of $+90°$ held across both signs of k_x, combined with an amplitude linear in $|k_x|$, gives $H \propto i|k_x|$, which is even. Differentiation requires $+90°$ for $k_x > 0$ and $-90°$ for $k_x < 0$. The phase computed at $k_x = 0$ itself carries no information in either channel, since $|H|$ there is 2.5×10^{-2} or below and the argument of a near-zero amplitude is not well conditioned.

S6 Polarization crosstalk

Table S3 gives the power leaked into the orthogonal polarization when the crystal axes are rotated in the plane by ϕ relative to the bar symmetry planes. Values are given both in the passband, 30 nm to the short-wavelength side of the TE operating wavelength, and at the operating wavelength itself. At the operating wavelength the co-polarized amplitude is small by construction, so the ratio there is large without the absolute leaked power being large; the passband values are the ones that bound the leakage in a device.

Table S3: Cross-polarized leakage against in-plane crystal misalignment ϕ. P_{co} and P_{cross} are transmitted powers in the zeroth order; the leaked fraction is $P_{\mathrm{cross}}/(P_{\mathrm{co}} + P_{\mathrm{cross}})$.

ϕ (deg)	Evaluated at	P_{co}	P_{cross}	Leaked fraction	$\frac{1}{4}\sin^2 2\phi$
0	Passband	2.44×10^{-1}	5.17×10^{-8}	2.1×10^{-7}	0
3	Passband	2.44×10^{-1}	1.35×10^{-6}	5.5×10^{-6}	0.0027
10	Passband	2.43×10^{-1}	1.39×10^{-5}	5.7×10^{-5}	0.0292
30	Passband	2.40×10^{-1}	9.52×10^{-5}	4.0×10^{-4}	0.1875
45	Passband	2.36×10^{-1}	1.37×10^{-4}	5.8×10^{-4}	0.2500
0	On resonance	2.79×10^{-4}	1.93×10^{-8}	6.9×10^{-5}	0
3	On resonance	2.77×10^{-4}	2.02×10^{-6}	7.2×10^{-3}	0.0027
10	On resonance	2.63×10^{-4}	2.30×10^{-5}	8.0×10^{-2}	0.0292
30	On resonance	1.49×10^{-4}	1.47×10^{-4}	5.0×10^{-1}	0.1875
45	On resonance	5.44×10^{-5}	1.94×10^{-4}	7.8×10^{-1}	0.2500

At $\phi = 0$ the leaked fraction in the passband is 2.1×10^{-7}, which is the numerical floor of the calculation and is consistent with the exact zero required by symmetry. The computed values lie well below the $\frac{1}{4}\sin^2 2\phi$ estimate quoted in the manuscript, so that estimate is conservative. Maintaining $|\phi| < 3°$ keeps the passband leakage below -50 dB.

S7 Sensitivity to geometry

Table S4 gives the resonance positions and quality factors for perturbations of the geometric parameters. These runs were carried out at 22 steps per wavelength rather than 40, so they should be read as shifts relative to the nominal row of the same table rather than as absolute values.

A ± 5 nm change in the gap moves the TE resonance by about 2.5 nm and the TM resonance by less than 1 nm. A 10 nm increase in bar width moves the TE resonance by 9.4 nm, so bar width is the tighter of the two lithographic tolerances. Opening the gap to 100 nm moves both resonances to longer wavelength and lowers the TE quality factor. Increasing the period to 650 nm raises both quality factors substantially, which

Table S4: Sensitivity of both channels to geometry. Computed at 22 steps per wavelength; read as shifts relative to the nominal row. The TE quality factor in the final row exceeds what this calculation can resolve and indicates a direction rather than a value.

Variation	TE λ_0 (nm)	TE Q	TM λ_0 (nm)	TM Q
Nominal	887.31	57.2	895.16	45.7
Gap +5 nm	889.78	56.9	895.82	45.5
Gap −5 nm	884.55	49.9	894.44	45.6
Bar width +10 nm	896.69	50.2	901.45	41.7
Gap 100 nm	906.01	46.1	900.01	42.7
Period 650 nm	910.95	$> 10^4$	952.92	116.2

is the direction to move in if a higher Q is wanted.

These runs vary the gap width, the bar width and the period. Variation in how completely the gap is filled along z was not modelled and is not covered by this table.

S8 Operation identification

Table S5 gives the fit statistics for the three candidate descriptions of each transfer function. The single-null high-pass form is

$$|H(k_x)| = \frac{|k_x|}{\sqrt{k_x^2 + \kappa^2}}. \tag{S2}$$

Table S5: Fit statistics for the transfer function of each channel. The high-pass fit uses the full swept range; the power-law fits use the stated momentum window.

Channel	Model	Parameter	R^2
TE	High-pass, κ (μm^{-1})	0.4356	0.939
TE	$\lvert H\rvert \propto \lvert k_x\rvert$, $\lvert k_x\rvert \le 0.5\ \mu$m^{-1}	1.656	−0.097
TE	$\lvert H\rvert \propto \lvert k_x\rvert$, $\lvert k_x\rvert \le 1.0\ \mu$m^{-1}	1.115	−0.172
TE	$\lvert H\rvert \propto k_x^2$, $\lvert k_x\rvert \le 1.0\ \mu$m^{-1}	1.248	−3.240
TM	High-pass, κ (μm^{-1})	1.1646	0.897
TM	$\lvert H\rvert \propto \lvert k_x\rvert$, $\lvert k_x\rvert \le 0.5\ \mu$m^{-1}	0.688	0.968
TM	$\lvert H\rvert \propto \lvert k_x\rvert$, $\lvert k_x\rvert \le 1.0\ \mu$m^{-1}	0.630	0.986
TM	$\lvert H\rvert \propto k_x^2$, $\lvert k_x\rvert \le 1.0\ \mu$m^{-1}	0.755	0.572

The TE channel is described by the high-pass form and is not described by either power law. The TM channel is described by both the high-pass form and, over the accessible momentum range, by a linear dependence on $|k_x|$, which follows from its cutoff lying near the top of that range.

S9 Image processing settings

The transfer function of each channel is computed at thirteen angles of incidence from 0° to 12° in 1° steps, giving thirteen samples of $H(k_x)$ on the branch $0 \le k_x \le 1.48\ \mu\text{m}^{-1}$. To apply it to an image, the object must be sampled so that its own spatial-frequency content falls inside that range. Setting the Nyquist frequency of the image equal to the largest computed k_x gives a pixel pitch

$$\Delta = \frac{\pi}{k_{x,\max}} = 2.12\ \mu\text{m}, \tag{S3}$$

which coincides with the diffraction limit of the swept numerical aperture, $\lambda/2\text{NA} = 880.8\ \text{nm}/(2\times 0.208) = 2.12\ \mu\text{m}$. A 512×512 image then covers a field of 1.09 mm. The transfer function is interpolated from the thirteen computed samples onto the image frequency axis on the branch $k_x \geq 0$ and mirrored onto $k_x < 0$, which the symmetry of Section S5 permits, and applied as a multiplicative filter along x. The filters are the computed amplitudes themselves and not the fitted forms of Table S5.

S10 Efficiency and numerical aperture

The swept momentum range is $|k_x| \leq 1.48\ \mu\text{m}^{-1}$, which corresponds to a numerical aperture of 0.208 at the TE operating wavelength and 0.211 at the TM operating wavelength. In the passband the TE channel transmits a power fraction of 0.70 and the TM channel 0.15. At $k_x = 0$ the residual transfer function amplitudes are 1.0×10^{-2} for TE and 2.5×10^{-2} for TM. The lower TM passband figure follows from the shape of the kernel, which rises from zero rather than approaching unity, and not from absorption, which Section S4 shows to be negligible here.

References

[1] Flexcompute Inc., *Tidy3D: GPU-accelerated finite-difference time-domain electromagnetic solver*, https://www.flexcompute.com/tidy3d/, 2026, Version 2.12.0.

[2] A. N. Toksumakov, D. V. Grudinin, G. A. Ermolaev, A. P. Tsapenko, A. A. Minnekhanov, K. V. Kravtsov, N. V. Pak, A. B. Mazitov, A. A. Vyshnevyy, A. S. Slavich, I. A. Kruglov, G. I. Tselikov, A. V. Arsenin, K. S. Novoselov and V. S. Volkov, *Advanced Materials*, 2026, **38**, e74228.
